# Inhibition of salty taste and sodium appetite by estrogens in spontaneously hypertensive rats

**DOI:** 10.3389/fphys.2023.1163568

**Published:** 2023-05-22

**Authors:** Emilson Donizete Pereira, Aline Aparecida Zenatti, José Vanderlei Menani, Laurival Antonio De Luca, Carina A. F. Andrade

**Affiliations:** Department of Physiology and Pathology, Dentistry School, São Paulo State University—UNESP, Araraquara, Brazil

**Keywords:** estradiol, palatability, hypertension, sodium, angiotensin II

## Abstract

Estrogen has a well-known effect of reducing salt intake in rats. This mini review focuses on recent findings regarding the interaction of estradiol with brain angiotensin II to control increased sodium palatability that occurs as a result of sodium appetite in spontaneously hypertensive rats.

## 1 Introduction

The ingestion of water and sodium is a necessary behavioral response for terrestrial animals to compensate for body fluid deficits. Sodium appetite occurs in response to prolonged extracellular dehydration or sodium depletion and is an innate and behaviorally motivated state influenced by learning, environmental conditions, and emotions (Denton et al., 1982; Schulkin, 1991).

Hormones play a significant role in inducing or constraining sodium intake in humans and rats (Santollo et al., 2023). This role involves the organization of future behavior from gestation and birth onward, in addition to the immediate effect of hormones on the ingestion of salt (Epstein, 1991; Mecawi et al., 2015). Angiotensin II (ANG II), for example, acts in the lamina terminalis of the third brain ventricle to produce sodium appetite, in collaboration with steroids that control fluid balance, such as aldosterone (Epstein, 1991; De Luca et al., 2010). In contrast, other hormones, some peptides and sex steroids among them, counteract the effect of ANG II (Mecawi et al., 2015). In addition to the effect on sodium consumption, estrogens also have a profound influence on hydromineral balance and cardiovascular parameters, and exert a beneficial effect in terms of control of arterial blood pressure in rats and humans (Hay et al., 2014; Vivas et al., 2014).

The objective of this mini-review is to highlight the role of taste in the inhibitory effect that estrogens exert on hypertonic NaCl intake as an expression of sodium appetite in rats. Thus, the neurobiology of sodium appetite is briefly reviewed, followed by description of the inhibitory effect of estrogens on sodium appetite, sodium preference, and sodium palatability in normotensive and hypertensive rats. Subsequently, we discuss the inhibitory effect of estrogens on sodium palatability in a model of sodium appetite produced by water deprivation and partial rehydration (WD-PR) in spontaneously hypertensive rats (SHR).

## 2 Neurobiology of sodium appetite

The control of sodium and water intake depends on forebrain and hindbrain areas involved in a circuitry that integrates facilitatory and inhibitory mechanisms (Johnson and Thunhorst, 2007; Formenti et al., 2013; Menani et al., 2014). Facilitatory mechanisms that produce water and sodium intake include ANG II and aldosterone. The action of the facilitatory mechanisms is counterbalanced by inhibitory mechanisms derived from visceral or brain sensory information (Johnson and Thunhorst, 2007; Menani et al., 2014).

The renin–angiotensin–aldosterone system (RAAS) is a key hormonal system activated by hypovolemia-related conditions (Fitzsimons, 1998). Central ANG II receptors are expressed in neuronal cells in several brain areas, such as the subfornical organ (SFO), organum vasculosum of the lamina terminalis (OVLT), paraventricular nucleus (PVN), median preoptic nucleus (MnPO), area postrema (AP), and nucleus of the solitary tract (NTS) (Lewis et al., 1986; McKinley et al., 1987; Allen et al., 1988; Allen et al., 1999). Among ANG II receptors, the most important for its dipsogenic effect are the AT1 receptors present in forebrain circumventricular organs, such as the SFO and OVLT (Fitzsimons, 1998).

Changes in blood pressure and volume are detected by receptors located in the aortic arch and carotid sinus (baroreceptors) and in the walls of large veins and the atria (volume receptors). Afferent signals from baroreceptors and cardiopulmonary receptors reach the brainstem via cranial nerves IX and X, with most of these nerve endings making their first synapse in the NTS (Johnson and Thunhorst, 2007). The NTS sends projections to the lateral parabrachial nucleus (LPBN), a pontine structure located dorsal to the superior cerebellar peduncle.

The LPBN acts as an important inhibitory mechanism for sodium intake control (for reviews, see Andrade et al., 2014; Menani et al., 2014). The LPBN also has important reciprocal connections with several other hindbrain and forebrain areas involved in the control of fluid and electrolyte balance (Ciriello et al., 1984; Fulwiler and Saper, 1984; Jhamandas et al., 1996; Johnson and Thunhorst, 2007). Signals from peripheral baroreceptors, cardiopulmonary volume receptors, taste receptors, or osmoreceptors ascend to the LPBN, and from this nucleus, they may influence the activity of forebrain areas important for the control of sodium intake.

Sodium appetite can be experimentally induced by various protocols. A slow-onset protocol involves subcutaneous injection of the diuretic furosemide (10 mg/mL/rat), followed by access to only water and sodium-deficient food for 24 h. This method promotes a loss of between 1.5 and 2.0 mEq of sodium and induces a consistent sodium appetite, which expresses or “matures” several hours after the injection of furosemide (Sakai et al., 1986; Rowland and Morian, 1999). An acute, rapid-onset sodium appetite can be induced by furosemide combined with a low dose of the angiotensin-converting enzyme blocker captopril (FURO + CAP). The FURO + CAP protocol advances the expression of sodium appetite to within 2 h (Thunhorst and Johnson, 1994; Thunhorst et al., 1998). The rapid induction of water and 0.3 M NaCl intake by FURO + CAP treatment results from mild hypotension - which unloads low-pressure volume receptors, combined with the action of ANG II in the brain (Fitts and Masson, 1989; Thunhorst et al., 1994; Thunhorst and Johnson, 1994).

The water deprivation–partial rehydration (WD-PR) protocol provides a method for production of clearly distinct thirst and sodium appetite, in which the rat feeds on regular laboratory chow, a sodium-excess diet relative to the animal’s need (De Luca et al., 2010). The WD-PR protocol involves a sequence of water deprivation with free access to regular laboratory chow (WD), followed by partial rehydration (PR), during which period the rat is allowed to drink only water to satiation. Drinking water to satiation during PR leads to the dilution of body fluids and partial recovery of the extracellular volume with sustained high systemic activation of the RAS (De Luca et al., 2002; De Luca et al., 2010). Thereafter, a sodium appetite two-bottle test (0.3 M NaCl and water) is conducted. Increased NaCl intake relative to water intake results in recovery of blood volume (De Luca et al., 2010). It also induces differential neuronal activity in the lamina terminalis and other brain areas relative to the preceding hydrated, WD, and WD-PR conditions (Pereira-Derderian et al., 2010; Dalmasso et al., 2015).

## 3 Estrogens and sodium appetite

Estrogens have a dual effect on sodium appetite in rats. On the one hand, their perinatal action confers a “female” character on offspring, thus being responsible for female sodium preference in adulthood (Epstein, 1991). On the other hand, estrogens limit the hydromineral fluid intake of females during estrus (Antunes Rodrigues and Covian, 1963; Danielsen and Buggy, 1980). This effect is associated with modulation of the hypothalamic secretion of inhibitory peptides, e.g., oxytocin, and downregulation of the brain renin–angiotensin system (RAS) (Mecawi et al., 2015; Almeida-Pereira et al., 2019).

Estrogens also modulate the local RAS in different organs. For example, in the kidney, estradiol (E2) decreases the activity of angiotensin-converting enzyme (ACE) and renin, as well as the mRNA expression of angiotensin II type 1 receptor (AT1R), but increases the mRNA expression of AT2R and the production of ANG-(1-7) (Shenoy et al., 2009; Brown et al., 2012; Bhatia et al., 2013). In the brain, E2 inhibits the mRNA expression of AT1R and ACE in the lamina terminalis (Kisley et al., 1999; Dean et al., 2006; Krause et al., 2006; Santollo and Daniels, 2015).

## 4 Sodium palatability

Humans, other, primates, and rats exhibit stereotyped orofacial reactions to different tastes applied directly to the oral cavity, and the evaluation of these reactions can be used as a behavioral measure of the hedonic value of taste: that is, taste palatability (Grill and Norgren, 1978; Berridge, 2000; Berridge and Kringelbach, 2015). Using this approach, previous studies have shown that the palatability of salty taste increases after sodium depletion (Berridge et al., 1984; Grill and Bernstein, 1988; Berridge and Schulkin, 1989). In other words, sodium depletion promotes an increase in the frequency of hedonic reactions and a decrease in aversive reactions in response to intraoral (IO) administration of NaCl solution, thus characterizing a hedonic shift in sodium palatability induced by sodium depletion (Berridge et al., 1984; Grill and Bernstein, 1988; Berridge and Schulkin, 1989).

A recent study from our laboratory with normotensive rats showed that antagonism of brain AT1R reduces hedonic responses and increases aversive responses to intraoral infusion of 0.3 M NaCl (Zenatti et al., 2021). Moreover, in the same strain of rats, increased endogenous production of brain ANG II sensitizes hedonic responses to intraoral infusion of the same solution (Fernandes et al., 2022).

## 5 Salt preference

Several studies have demonstrated an increase in preference for sodium in humans undergoing alterations in fluid and electrolyte balance, such as administration of diuretics accompanied by a low-sodium diet and water deprivation, as well as sweating, hemodialysis, and gestation (Beauchamp et al., 1990; Takamata et al., 1994; Leshem and Rudoy, 1997; Duffy et al., 1998). However, excessive NaCl intake is a risk factor for chronic diseases, including hypertension. Limited sodium intake (less than 2 g/day) is recommended to control blood pressure and reduce the risk of cardiovascular diseases (He and Macgregor, 2012; World Health Organization, 2016); this recommendation is difficult to follow because dietary NaCl increases food palatability (Keast and Breslin, 2003; Liem et al., 2011). Moreover, increased salt preference may be associated with pathological conditions. This can be observed in medicated, compensated patients undergoing chronic treatment for heart failure (de Souza et al., 2012). The same is observed in patients with primary aldosteronism, who show reduced taste perception of NaCl (Adolf et al., 2021). This favors a higher dietary intake, which in turn accelerates the progress of arterial hypertension (Adolf et al., 2021).

Investigations of preference for salt in hypertension can benefit from the use of animal models of palatability.

## 6 Sodium intake and sodium palatability in spontaneously hypertensive rats

The spontaneously hypertensive rat (SHR) is a traditional model for the study of essential hypertension (Elmarakby and Sullivan, 2021). In addition to high blood pressure, the adult SHR exhibits enhanced sodium intake, as measured by ingestion of NaCl solution (for early reviews, see Ely et al., 2000; Di Nicolantonio et al., 2004). This intense NaCl intake arises from a hyperactive brain RAS rather than from altered fluid–electrolyte balance (DiNicolantonio et al., 1982; Di Nicolantonio et al., 2004). Brain injection of captopril, an ACE inhibitor, reduces daily NaCl intake in the SHR (DiNicolantonio et al., 1982). Accordingly, the SHR is suitable for modeling of excess sodium intake in the human context under different hydration conditions and difficulty in controlling arterial pressure (Takamata et al., 1994; Millard-Stafford et al., 2012; Lucia and Curtis, 2018; Mill et al., 2019; Minegishi et al., 2020; Orysiak et al., 2022; Zhang et al., 2022).

As predicted, the SHR exhibits higher hypertonic NaCl intake than normotensive strains in response to WD-PR (Pereira-Derderian et al., 2010). The SHR also exhibits a different pattern of encephalic c-Fos expression, particularly in the lamina terminalis, in response to WD-PR (Pereira-Derderian et al., 2010). In addition, the lamina terminalis of this strain expresses more Fos-IR in response to either brain or systemic infusions of ANG II (Rowland et al., 1995; Blume et al., 1997). Finally, inhibition of brain AT1R-mediated ERK1/2 activation reduces 0.3 M NaCl intake and the ANG II pressor response in the SHR (Andrade-Franze et al., 2020). Taken together, these findings suggest that the high intake of hypertonic NaCl exhibited by the SHR in response to WD-PR results from hyperactive brain RAS activity, particularly in the lamina terminalis.

By investigating sodium palatability in male SHRs and normotensive rats under euhydrated and dehydrated conditions, we have previously shown that SHRs also have enhanced palatability for NaCl taste, as measured by an increased number of hedonic versus aversive responses to IO infusion (1 mL/1 min) of 0.3 M NaCl in a taste reactivity test, irrespective of their body fluid status (Pereira et al., 2021).

## 7 Discussion

Considering that estrogens exert an inhibitory influence on sodium intake and that SHRs constitute a model for the study of increased sodium intake and palatability in the context of essential hypertension, we aimed to investigate sodium palatability in female SHRs in comparison to normotensive rats. In this section, we compare the findings of recent studies with female SHRs (Pereira et al., 2019; Pereira et al., 2022) to those of a study with male SHRs (Pereira et al., 2021), discussing the possible mechanisms involved in the effects of estrogen on sodium palatability in SHRs.

**FIGURE 1:**
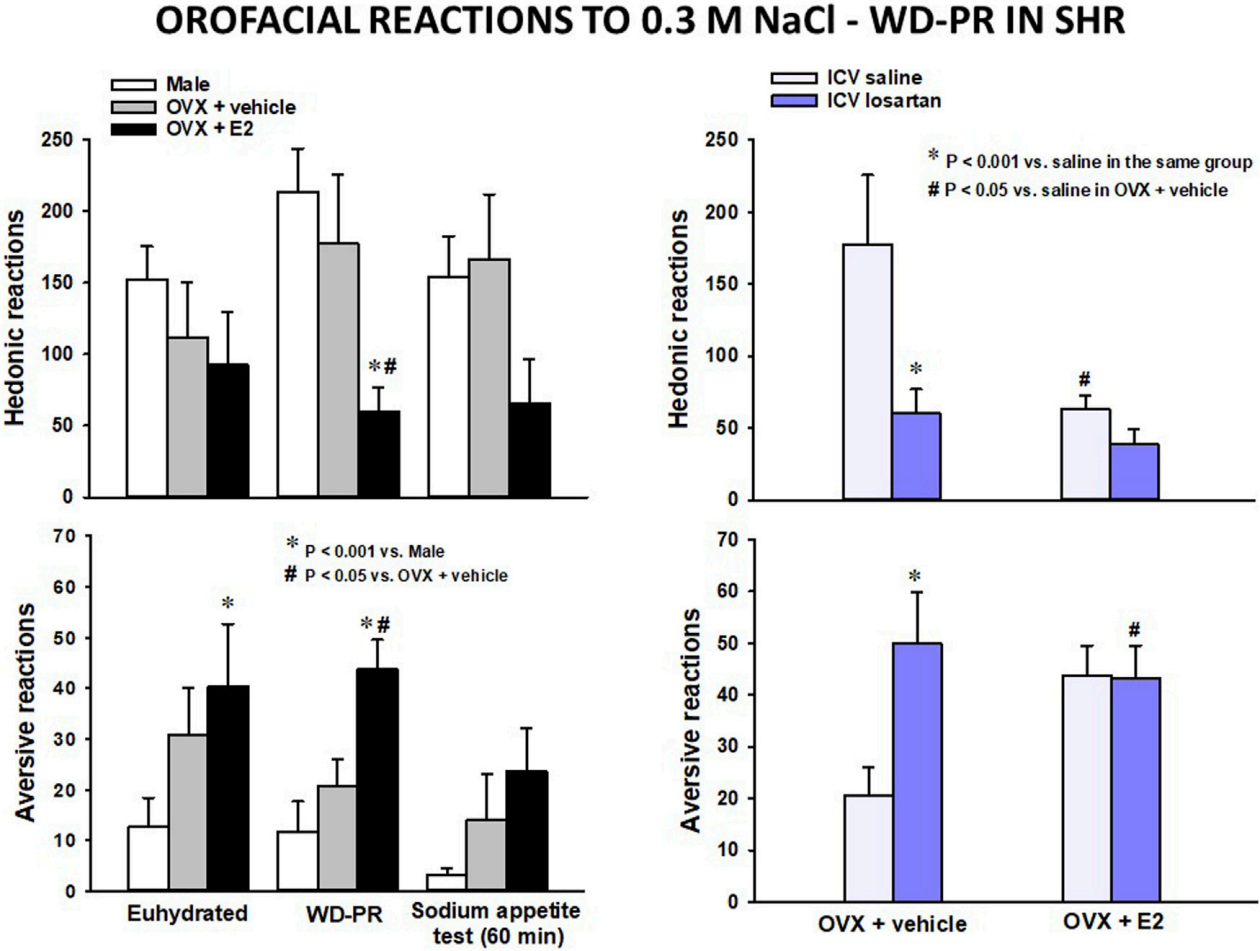
Adapted from our previous studies (Pereira et al., 2021; Pereira et al., 2022) with permission from Appetite and Hormones and Behavior. Left: the number of hedonic (top) and aversive (bottom) responses to IO infusion of 0.3 M NaCl in a euhydrated condition, after WD-PR and following the sodium appetite test in female and male SHRs submitted to this protocol. Right: the number of hedonic (top) and aversive (bottom) responses to IO infusion of 0.3 M NaCl after WD-PR in ovariectomized (OVX) SHRs treated with vehicle or estradiol (E2, 10 μg/kg/rat) s.c. combined with saline or losartan (100 μg/μL) ICV. Results are presented as means ± SEM. Differences were analyzed by two-way ANOVA and the Student–Newman–Keuls test.

Using hormone replacement therapy (Sampson et al., 2012; Tazumi et al., 2016), we have investigated the role of E2 in salt taste in ovariectomized female SHRs “clamped” in simulated physiological estrus (Pereira et al., 2019). Under the WD-PR protocol, E2 exerted a strong effect (>50% alteration) on the occurrence of hedonic (reduced) and aversive (increased) orofacial reactions to intraoral infusion of 0.3 M NaCl in SHRs (Figure 1, left). There was also a reduction of approximately 75% in 0.3 M NaCl intake in a subsequent sodium appetite test (not shown). In contrast, ovariectomized (OVX) control SHRs that received no estogens exhibited orofacial reactions comparable to those of male SHRs (Figure 1, left). OVX SHRs also ingested the same amount of 0.3 M NaCl as male SHRs (not shown). Recall that male SHRs exhibit not only notably high 0.3 M NaCl intake but also strong orofacial hedonic reactions and a low rate of aversive reactions to IO of this solution, even in a euhydrated condition, relative to normotensive strains (Pereira-Derderian et al., 2010; Pereira et al., 2021). As shown in Figure 1 (left, bottom), E2 significantly increased the occurrence of aversive reactions in SHRs in a euhydrated condition.

In a follow-up investigation of responses to WD-PR (Pereira et al., 2022), intracerebroventricular (ICV) injection of losartan (an AT1R antagonist) was found to have no effect on orofacial reactions to IO of 0.3 M NaCl in E2-treated SHRs (Figure 1, right). In contrast, losartan strongly reduced hedonic reactions and increased aversive reactions in OVX SHRs. ICV losartan also abolished 0.3 M NaCl intake in a sodium appetite test among OVX and E2-treated SHRs (not shown). Finally, and similarly to the outcome in the brains of normotensive rats (Krause et al., 2006), E2-treated SHRs exhibited reduced hypothalamic mRNA expression of AT1R (Pereira Jr. et al., 2022).

The SHR/WD-PR model, along with data from a normotensive strain, provides fresh evidence for the roles of brain ANG II and estrogen in emotion-based control of behavior in relation to salty taste (Pereira et al., 2019; Pereira et al., 2021; Zenatti et al., 2021; Fernandes et al., 2022; Pereira Jr. et al., 2022). We conclude that alteration of salt palatability is a crucial key in understanding why estrogen inhibits NaCl intake in SHRs. The underlying mechanism resides in the brain, involving the attenuation of ANG II-induced increases in salt palatability. The extent to which this attenuation depends on the modulation of salt taste afferents (Shigemura et al., 2013; Martin and Sollars, 2017) is open to further research.

**FIGURE 2:**
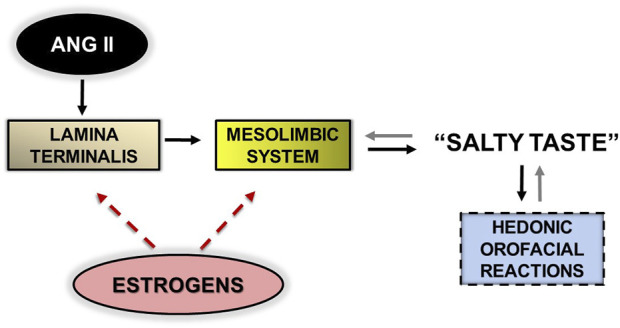
Flow diagram representing the sequence of brain events triggered by the action of angiotensin II (ANG II) on the lamina terminalis, leading to “pleasure” in experience of salty taste, expressed by hedonic, orofacial motor somatic reactions (*solid black arrows*). The presence of salt in the oral cavity feeds back sensory information (via the brainstem; not shown) to the mesolimbic system to control hedonic responses (*solid gray arrows*). Estrogen activates brain mechanisms that attenuate this flow, thereby opposing the production of hedonic reactions to salty taste; it acts on the lamina terminalis (or adjacent areas; see text) and on the mesolimbic system (*red dotted arrows*).

Orofacial reactions to taste correspond directly to the consummation of the motivation to ingest salt, a behavior modulated by the mesolimbic system (Berridge, 2018; Hsu et al., 2018). Estrogens modulate neuronal activity in the preoptic/hypothalamic area in connection with mesolimbic dopaminergic transmission (Champagne and Curley, 2016). As a final note, Figure 2 illustrates how estrogens interact with brain ANG II to inhibit salty taste, thereby inhibiting sodium appetite.
